# Anti-Arthritic Activity of Bartogenic Acid Isolated from Fruits of *Barringtonia racemosa* Roxb. (Lecythidaceae)

**DOI:** 10.1093/ecam/nep148

**Published:** 2011-02-20

**Authors:** Kalpesh Ramdas Patil, Chandragouda Raosaheb Patil, Ramchandra Baburao Jadhav, Vallabh Krishnalal Mahajan, Prabhakar Raosaheb Patil, Pradeep Sampatrao Gaikwad

**Affiliations:** ^1^Department of Pharmacology, R.C. Patel Institute of Pharmaceutical Education and Research, Karvand Naka, Shirpur, Maharashtra, India; ^2^Navodaya Medical College, Raichur, Karnataka, India

## Abstract

The fruits of *Barringtonia racemosa* are prescribed in the ayurvedic literature for the treatment of pain, inflammation and rheumatic conditions. In present investigation, activity guided isolation of bartogenic acid (BA) and its evaluation in the Complete Freund's Adjuvant (CFA)-induced arthritis in rats is reported. Among the various extracts and fractions investigated preliminarily for carrageenan-induced acute inflammation in rats, the ethyl acetate fraction displayed potent anti-inflammatory activity. Large-scale isolation and characterization using chromatography and spectral study confirmed that the constituent responsible for the observed pharmacological effects was BA. Subsequently the BA was evaluated for effectiveness against CFA-induced arthritis in rats. The results indicate that at doses of 2, 5, and 10 mg kg^−1^ day^−1^, p.o., BA protects rats against the primary and secondary arthritic lesions, body weight changes and haematological perturbations induced by CFA. The serum markers of inflammation and arthritis, such as C-reactive protein and rheumatoid factor, were also reduced in the BA-treated arthritic rats. The overall severity of arthritis as determined by radiological analysis and pain scores indicated that BA exerts a potent protective effect against adjuvant-induced arthritis in rats. In conclusion, the present study validates the ethnomedicinal use of fruits of *B. racemosa* in the treatment of pain and inflammatory conditions. It further establishes the potent anti-arthritic effects of BA. However, additional clinical investigations are needed to prove the efficacy of BA in the treatment of various immuno-inflammatory disorders.

## 1. Introduction

The current treatment of rheumatoid arthritis is intended to minimize the associated pain and inflammation using non-steroidal anti-inflammatory drugs (NSAIDs) as well as to decelerate the progress of the disease by using disease-modifying anti-rheumatic drugs (DMARDs). DMARDs suppress the immunological processes involved in the progression of rheumatoid arthritis. Drugs that have the effects of both DMARDs and NSAIDs may be more effective in the treatment of rheumatoid arthritis, but there is a scarcity of such drugs acting through multiple mechanisms. Hence, the treatment of rheumatoid arthritis involves the combined use of NSAIDs and DMARDs [1]. Due to chronic nature of rheumatoid arthritis, advanced age of the patients and adverse reactions of the NSAIDs and DMARDs, the arthritic patients tend to search for alternative treatments that are effective and less toxic and reduce the pill burden. Hence, they commonly prefer complementary and alternative medicines [2].

In Indian traditional medicines, ayurvedic literature describes potions containing parts of certain plants for treating painful and inflammatory conditions like arthritis. *Barringtonia racemosa* Roxb. (Lecythidaceae) is one such plant [3, 4]. The extracts prepared from different parts of this plant have been reported to have various biological activities such as anti-tumor, anti-nociceptive, *α*-glucosidase inhibitory, anti-bacterial and anti-fungal activities [5–9]. Recently an extract of the leaves of *B. racemosa* has been reported to possess anti-inflammatory and anti-oxidant activity [10]. The observed activities have been correlated with the lycopene present in *B. racemosa* leaves.

Several diterpenoids and triterpenoids have also been isolated from various parts of *B. racemosa* and it has been proposed that some of these are implicated in the biological activities of the plant. The ethnomedicinal use of the fruits of *B. racemosa* in arthritic disorders, however, has not been systematically investigated so far. Furthermore, the phytochemical moiety responsible for these effects is not yet known. Therefore, the present study was designed to determine the anti-arthritic activity of bartogenic acid (BA), a pentacyclic triterpenoid isolated from the fruits of *B. racemosa*, in the Complete Freund's Adjuvant (CFA)-induced arthritis model in rats.

## 2. Methods

### 2.1. Plant Material and Chemicals

Fruits of *B. racemosa* were purchased from the local market. Dr P.S.N. Rao of the Botanical Survey of India, Pune, India, authenticated a specimen (voucher no 74843). A sample of an authentic marker of BA was generously provided by Dr Mangala Gowri, Senior Scientist, Indian Institute of Chemical Technology (IICT), Hyderabad, India, as a gift. A diclofenac sodium (DCS) sample was received from Cipla Ltd, Goa, India, as a gift. CFA was purchased from Sigma Aldrich, USA. C-reactive protein (CRP) and Rhelax rheumatoid factor (RF) kits were purchased from Agappe Diagnostics Pvt. Ltd, Kerala, India, and Tulip Diagnostics Pvt. Ltd., Goa, India, respectively. Anesthetic ether was obtained from Loba Chemicals, Mumbai. The other chemicals and solvents used in the extraction, fractionation and chromatographic separations were of analytical grade.

### 2.2. Extraction, Isolation and Characterization of BA

**Figure 1:**
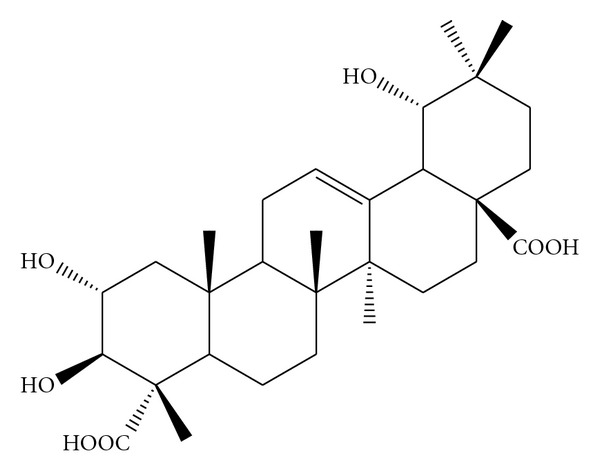
Chemical structure of BA (2*α*,3*β*,19*β*-trihydroxyolean-12-en-23-28-dioic acid).

Hexane, ethanol and methanol extracts were obtained by cold maceration. The methanol extract displayed the most potent anti-inflammatory activity in the carrageenan-induced paw inflammation model in rats. Hence this extract was further fractionated into *n*-butanol and ethyl acetate fractions. When compared with the same model of acute inflammation, the ethyl acetate extract had a more potent effect. Hence it was used for further fractionation on a silica gel chromatography column, according to the procedure described by Mangala et al. [9]. The pure compound, obtained from large-scale extraction and isolation, was characterized by comparing it with the authentic marker as well as by comparing its FT-IR, proton NMR and LC-MS spectral data with those in the literature [9]. The chromatographic and spectral data are consistent with those of a previous study [9], which confirms that the isolated compound was BA (Figure 1).

### 2.3. Animals and Experimental Design

Wistar rats of either sex (100–150 g weight) were used. The animals were maintained in plastic cages at 22 ± 2°C with free access to pellet food and water. The experimental protocols were approved by the Institutional Animal Ethical Committee constituted as per the rules of the Committee for the Purpose of Control and Supervision of Experiments on Animals, India (CPCSEA), laid down by the Government of India (Regd. No. 651/02/C/CPCSEA).

### 2.4. CFA-Induced Arthritis in Rats

Each treatment group contained six Wistar rats. The rats were randomly divided into five groups: CFA control, BA (2 mg kg^−1^ day^−1^, p.o.), BA (5 mg kg^−1^ day^−1^, p.o.), BA (10 mg kg^−1^ day^−1^, p.o.) and DCS (5 mg kg^−1^ day^−1^, p.o.). Arthritis was induced by injection of CFA according to a method described by Kumar et al. [11]. In brief, on day 0, 100 *μ*L of CFA containing heat-killed and dried *Mycobacterium tuberculosis* (strain H37Ra, ATCC-25177) was injected into the paw of the right hind limb of each rat.

### 2.5. Evaluation of the Severity of Arthritis

The primary and secondary lesions, that is, paw volumes of injected and non-injected paws, were measured using a digital plethysmometer (UGO Basile 7140, Italy), after which adjuvant was administered. The lesions were measured again on the 7th, 14th, and 21st days after injection of the adjuvant [12, 13]. During the experimental period, the body weight was measured using a digital weighing balance every 3rd day after adjuvant injection. The severity of arthritis was recorded by a blinded observer using the visual arthritis scoring systems described by Kumar et al. and Laird et al. [11, 14]. The arthritis score ranged from 0 to 4; where 0 indicates the least but definite swelling and 4 represents the maximum swelling. This scoring system involves observations of all four paws and giving a separate score for each limb. Scores were assigned for evaluation of the pain associated with the arthritis as shown in Table 1. 

Haematological parameters were evaluated using routine laboratory methods. The level of serum CRP and RF was determined using commercial kits according to the manufacturers' instructions.

### 2.6. Radiological Analysis

On day 21, animals were anesthetized with anesthetic ether. Radiographs of the adjuvant-injected hind paws were taken with a X-ray instrument (GE-525 DX, USA) Fuji computerized radiographic systems (Japan). The film focus distance was 60 inches and the machine was operated at 43 kV peak, 2 mA. The X-ray image of the adjuvant-injected limb of each rat was evaluated for radiographic changes. As reported previously [14–16] the radiological alterations were arbitrarily graded between 0 and 4 according to the severity of the swelling of the soft tissue around the joints of the hind paws, periarticular bone resorption, periarticular bone erosion and narrowing of the joint space. At the end of the study, the animals were sacrificed with an overdose of anesthetic ether. The thymus and spleen of all the animals were removed and weighed [17, 18].

### 2.7. Statistical Analysis

The results are expressed as the mean ± SEM for the parametric data sets and as the median (minimum, maximum) for the non-parametric data sets. The significance of the difference was evaluated by one-way ANOVA followed by Dunnett's multiple comparisons test for normal data and by the Kruskall-Wallis test followed by Dunn's multiple comparison test for the scored data. Data were considered statistically significant if *P* < .05.

## 3. Results

### 3.1. BA Reduced Primary and Secondary Arthritic Lesions in Rats

Observations such as the paw volumes, body weight and arthritis scores were recorded on the 7th, 14th, and 21st days after adjuvant injection. The CFA-induced arthritis control group showed signs of arthritis development, as seen by the increase in the paw volumes in both CFA-injected and CFA-non-injected paws, which indicates primary and secondary arthritic lesions. Other indications, such as a decreased body weight and alterations in the arthritis scores, also showed induction of arthritis in the CFA-treated control group rats.

The assessment made on the 21st day showed that the DCS and BA treatments had significantly reduced the adjuvant-induced primary and secondary lesions in the respective treatment groups as compared with the CFA control group (Table 2). It is noteworthy that the reduction in the secondary lesions was comparable in the DCS-treated and BA 5 mg/kg treated groups.

### 3.2. BA Improved the Body Weight Gain and Decreased Spleen and Thymus Weights in Arthritic Rats

The average gain in the body weight on day 21 as compared with the initial body weight in each treatment group has been given in Table 3. The rats in the CFA control group gained less body weight as compared with the BA- and DCS-treated groups. This effect on the body weight was clearly evident even at the lowest tested dose of 2 mg/kg of BA.

The weights of the spleen and thymus recorded after sacrificing the rats on day 21 were significantly reduced in the BA-treated group. The 10 mg/kg dose of BA caused a greater reduction in the thymus weight than did the DCS (5 mg/kg) treatment.

### 3.3. Haematological Alterations in CFA Rats were Normalized after Treatment with BA

The CFA-induced haematological perturbations, such as an increase in the WBC count, a decreased RBC count, a decreased hemoglobin (Hb) count and an increased erythrocyte sedimentation rate were also favorably altered by BA treatment (Table 4).

### 3.4. The Levels of CRP and RF were Suppressed by BA Treatment

The serum CRP and RF are markers of systemic inflammation and antibody production against the injected adjuvant. High levels of serum CRP (8.8 mg/dL) and serum RF (73 IU/mL) were observed in the CFA control group rats. The BA and DCS treatments reduced the increase in the levels of both CRP and RF in the serum (Table 4). The effects of BA were dose-dependent, and the 10 mg/kg dose of BA and 5 mg/kg dose of DCS had equipotent effects in decreasing the serum RF levels.

### 3.5. Pain Scores were Normalized in the BA-Treated Groups

BA treatment favorably affected the pain scores, indicating a significant decrease in the pain associated with the adjuvant-induced arthritis (Table 5). All the estimated pain scores, including the flexion pain test score, mobility score and stance score were significantly altered in DCS-treated and BA (10 mg/kg)-treated rats. The reduction in the mobility score was greater in the BA (10 mg/kg)-treated group as compared with the DCS-treated group.

### 3.6. BA Protected the Rats from CFA-Induced Radiographic Changes

**Figure 2:**
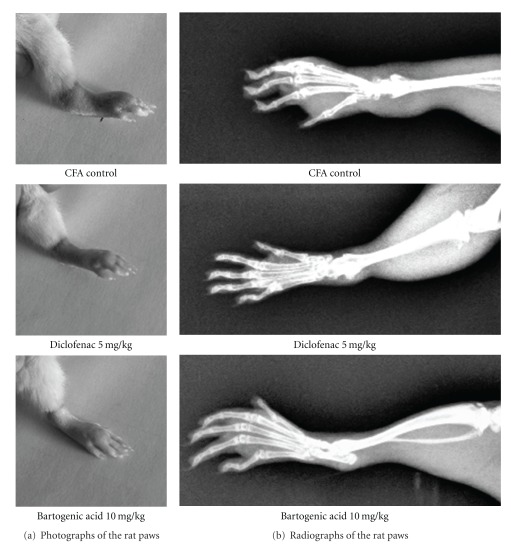
Photographic and radiographic analysis of CFA-induced arthritis in rats. (a) Photograph of the right hind paw taken 14 days after CFA injection and (b) Radiographic analysis of the same right hind paws at Day 21 after CFA injection.

Figure 2(a) shows representative photographs of the tarsotibial joint swelling of the right hind paws of rats from different groups on the 14th day after CFA injection.  Figure 2(b) shows X-ray radiographs of the same paws taken on the 21st day. It is clearly observed in the X-rays that the soft tissue swelling around the joints, periarticular bone resorption, periarticular bony erosions and joint space narrowing in the rats treated with BA have been protected from the CFA-induced arthritis-related joint changes.

## 4. Discussion

CFA-induced arthritis is the most widely used chronic test model in which the clinical and pathological changes are comparable with those seen in human rheumatoid arthritis [19, 20]. Chronic inflammation in the CFA model is manifested as a progressive increase in the volume of the injected paw. It is noteworthy that the inhibitory effect of BA (10 mg kg^−1^ day^−1^) on the volume of the injected paw was comparable with that of DCS (5 mg kg^−1^ day^−1^) (Table 2).

CFA-induced polyarthritis is associated with an immune-mediated inflammatory reaction and the rat is unique in developing polyarthritis after CFA treatment [16]. The initial reaction of edema and soft-tissue thickening at the depot site in this model is caused by the irritant effect of the adjuvant, whereas the late-phase arthritis and flare in the injected foot are presumed to be immunologic events [21]. The appearance of secondary lesions, that is, non-injected paw swelling is a manifestation of cell-mediated immunity. The suppression of such secondary lesions by a drug shows its immunosuppressive activity [12, 13]. BA effectively reduced the secondary lesions in arthritic rats (Table 2). Moreover, this effect of BA was more potent than that of diclofenac. This reveals potent suppression by BA of cell-mediated immunity in arthritic rats. Similarly, it reduced the arthritic score and secondary paw swelling. A selective reduction in the arthritis score distinguishes the immunosuppressive effects of a drug from its anti-inflammatory effects [15]. The reduction of the arthritis score by BA as observed in our study indicates a possible immunosuppressant effect (Table 5). The significant reduction of the thymus weight in the BA-treated groups further supports this observation.

CFA-induced arthritis in rats is associated with an increase in the plasma levels of RF and CRP [22, 23]. The treatment with BA significantly reduced the levels of these biomarkers of inflammation and autoimmune stimulation in the treated rats (Table 4).

Other miscellaneous information related to the pathology of arthritis that has been obtained during this study includes radiographic examination of the paws, haematological parameters, body weight changes, organ weight changes and paw withdrawal latency. The radiographic observations of the rats show that the treatment with BA and DCS inhibited the arthritis-associated joint changes (Figure 2). In the DCS- and BA-treated groups there was restoration of the body weights of the rats. A report by Patil et al.[24] suggests that the decrease in the body weight during inflammation is due to deficient absorption of nutrients through the intestine and that treatment with anti-inflammatory drugs normalizes the process of absorption. The evident restoration of the body weight of rats in the BA- and DCS-treated groups may involve improvement of intestinal absorption of the nutrients and a reduction in the distress caused by the severity of the arthritis.

It has been reported that a moderate rise in the WBC count occurs in arthritic conditions due to an IL-1B-mediated rise in the respective colony-stimulating factors. The present study reveals that BA and DCS treatments tend to normalize the WBC count. In addition to this, other characteristic haematological alterations such as the decreased Hb count and increased erythrocyte sedimentation rate [12] were also restored by the BA and DCS treatments (Table 4). It is proposed that the reduction in the Hb count during arthritis results from reduced erythropoietin levels, a decreased response of the bone marrow erythropoietin and premature destruction of red blood cells. Similarly, an increase in the ESR is attributed to the accelerated formation of endogenous proteins such as fibrinogen and *α*/*β* globulin, and such a rise in the ESR indicates an active but obscure disease process [18]. Thus, the reduction in the ESR and increase in the Hb count brought about by BA treatment further support its anti-arthritic effect.

The reduction in spleen weight and increase in thymus weight are related to a stimulatory effect on the immune system [25]. The observed decrease in the spleen and thymus weights in the BA-treated rats indicates alterations in the cell populations in these organs, which are related to the immune function. Dexamethasone produces a reduction in spleen and thymus weights that can be attributed to its anti-proliferative action. BA also displayed similar effects on these organs. Although these observations indicate an immunosuppressant activity of BA, direct evidence needs to be generated to confirm the immunosuppressant effect.

Arthritis is an inflammatory condition of the bone joints that is associated with hyperalgesia and functional impairment. The hyperalgesia associated with arthritis is mediated through prostaglandins and other endogenous mediators [26]. In the present study, the effect of BA on arthritis-induced hyperalgesia was evaluated by visually attributed arthritic scores and the dorsal flexion pain test. BA effectively increased the pain threshold and reduced the flexion pain test score (Table 5). Furthermore, the mobility and stance scores, which are commonly used to assess the functional impairment in arthritis were determined. BA treatment lowered the mobility score and improved the stance score, indicating a reduction in pain (Table 5). Though actual quantification of the mediators of pain was not performed in this study, it is proposed that BA significantly affects the levels/effects of such endogenous pain mediators [11].

**Figure 3:**
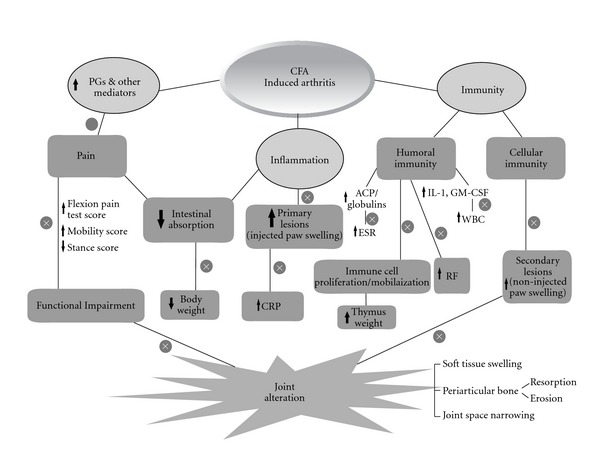
Anti-arthritic activity of BA. Cross mark indicates inhibition of CFA induced pathological changes in rats by Bartogenic acid. GM-CSF, granulocyte macrophage colony stimulating factor; ACP, acute phase proteins.

It is now recognized that single target- and single molecule-based drug development has been less relevant in many chronic diseases because these diseases involve multiple organ systems and interdependent etiological factors. Even mainstream drug discovery is now deviating from such a single molecule and single target approach to combinations and multiple-target approaches [27]. The treatment of a disease like arthritis is expected to address the alterations in the multiple mediators and/or their effects to derive clinical benefits. As evident from the results of the present study, a plant-derived triterpenoid, BA appears to exert beneficial effects on multiple pathological manifestations of CFA-induced arthritis in rats (Figure 3). Therefore this molecule may prove to be of clinical value if systematically investigated further. In addition, the present study validates the ethnomedicinal use of fruits of *B. racemosa* in the treatment of pain and inflammatory conditions.

## Figures and Tables

**Table 1:** Scoring system for evaluation of the pain associated with adjuvant induced arthritis.

Score	Flexion pain test score	Mobility score	Stance score
0	No squeaking and no leg withdrawal	Normal	—
1	Either squeaking or leg withdrawal	Limping	Paw lifted continuously
2	Both squeaking and leg withdrawal	Walking with difficulty	Paw touching but with no weight bearing
3	—	Walking without touching the injected paw	Some weight bearing on the paw
4	—		Normal

**Table 2:** Primary and secondary arthritic lesions at 21st day in CFA-induced arthritis in rats.

Treatment	Primary lesions (% rise in injected paw volume)	Secondary lesions (% rise in non-injected paw volume)
CFA control	146 ± 4.4	19 ± 0.67
Diclofenac (5 mg kg^−1^ day^−1^, p.o.)	83 ± 4.4**	13 ± 0.63**
BA (2 mg kg^−1^ day^−1^, p.o.)	113 ± 7.6**	15 ± 0.65**
BA (5 mg kg^−1^ day^−1^, p.o.)	109 ± 3.3**	12 ± 0.53**
BA (10 mg kg^−1^ day^−1^, p.o.)	91 ± 2.7**	11 ± 0.57**

Data represented in mean ± SEM (*n* = 6).

**P < .01, compared with control group.

**Table 3:** Body and organ weights changes in CFA-induced arthritis in rats.

Group	Body weight gain (g)	Thymus weight (g)	Spleen weight (g)
CFA control	24 ± 1.1	0.19 ± 0.022	1.1 ± 0.12
Diclofenac	41 ± 1.7**	0.1 ± 0.0074**	0.95 ± 0.042
BA (2 mg kg^−1^ day^−1^, p.o.)	37 ± 1.6**	0.12 ± 0.011**	0.96 ± 0.085
BA (5 mg kg^−1^ day^−1^, p.o)	36 ± 0.96**	0.12 ± 0.013**	0.84 ± 0.048*
BA (10 mg kg^−1^ day^−1^ p.o)	39 ± 1.1**	0.085 ± 0.009**	0.74 ± 0.085**

Data represented in mean ± SEM (*n* = 6).

*P < .05, **P < .01, compared with control group.

**Table 4:** Alterations in hematological parameters, CRP and RF in CFA-induced arthritis in rats.

Group	RBC (×10^6^/mm^3^)	WBC (×10^3^/mm^3^)	ESR (mm/h)	Hb (mg%)	CRP (mg/dL)	RF (IU/mL)
CFA control	7.3 ± 0.2	13 ± 0.4	14 ± 0.3	12 ± 0.4	8.8 ± 0.8	73 ± 6.7
Diclofenac (5 mg kg^−1^ day^−1^, p.o.)	9.2 ± 0.2**	5.5 ± 0.2**	10 ± 0.2**	15 ± 0.3**	1.8 ± 0.3**	23 ± 3.3**
BA (2 mg kg^−1^ day^−1^, p.o.)	8 ± 0.3	7.5 ± 0.3**	12 ± 0.2**	13 ± 0.5	4.4 ± 0.4**	37 ± 3.3**
BA (5 mg kg^−1^ day^−1^, p.o.)	9 ± 0.2**	7.2 ± 0.3**	13 ± 0.3	15 ± 0.4**	2.2 ± 0.2**	33 ± 4.2**
BA (10 mg kg^−1^ day^−1^, p.o.)	9.8 ± 0.2**	5.6 ± 0.2**	11 ± 0.3**	16 ± 0.3**	1.4 ± 0.2**	23 ± 3.3**

Data represented in mean ± SEM (*n* = 6).

**P < .01, compared with control group.

**Table 5:** Changes in various pain test scores in CFA-induced arthritis in rat.

Group	Arthritis score	Flexion pain test score	Mobility score	Stance score
CFA control	12 (10, 13)	3 (2, 3)	3 (2, 3)	1 (1, 2)
Diclofenac (5 mg kg^−1^ day^−1^, p.o.)	4.5 (3, 6)**	1 (1, 2)**	2 (1, 2)*	3 (2, 3)**
BA (2 mg kg^−1^ day^−1^, p.o.)	10 (9, 11)	2 (1, 3)	2 (2, 3)	2 (1, 3)
BA (5 mg kg^−1^ day^−1^, p.o)	9 (6, 11)	2 (1, 2)	2 (1, 3)	2 (2, 3)
BA (10 mg kg^−1^ day^−1^, p.o)	6 (4, 8)**	1.5 (1, 2)*	1.5 (1, 2)**	3 (2, 3)**

Data represented in median (minimum, maximum), *n* = 6.

*P < .05, **P < .01, compared with control group.
